# Household conditions, COVID-19, and equity: Insight from two nationally representative surveys

**DOI:** 10.21203/rs.3.rs-3129530/v1

**Published:** 2023-07-06

**Authors:** Nathan Kim, Elyssa Anneser, MyDzung T. Chu, Kimberly H. Nguyen, Thomas J. Stopka, Laura Corlin

**Affiliations:** Tufts University; Tufts University; Tufts Medical Center; Tufts University; Tufts University; Tufts University

**Keywords:** COVID-19 diagnosis, COVID-19 vaccination, household size, household conditions, environmental justice

## Abstract

**Background:**

With people across the United States spending increased time at home since the emergence of COVID-19, housing characteristics may have an even greater impact on health. Therefore, we assessed associations between household conditions and COVID-19 experiences.

**Methods:**

We used data from two nationally representative surveys: the Tufts Equity Study (TES; n = 1449 in 2021; n = 1831 in 2022) and the Household Pulse Survey (HPS; n = 147,380 in 2021; n = 62,826 in 2022). In the TES, housing conditions were characterized by heating/cooling methods; smoking inside the home; visible water damage/mold; age of housing unit; and self-reported concern about various environmental factors. In TES and HPS, household size was assessed. Accounting for sampling weights, we examined associations between each housing exposure and COVID-19 outcomes (diagnosis, vaccination) using separate logistic regression models with covariates selected based on an evidence-based directed acyclic graph.

**Results:**

Having had COVID-19 was more likely among people who reported poor physical housing condition (odds ratio [OR] = 2.32; 95% confidence interval [CI] = 1.17–4.59; 2021), visible water damage or mold/musty smells (OR = 1.50; 95% CI = 1.10–2.03; 2022), and larger household size (5+ versus 1–2 people; OR = 1.53, 95% CI = 1.34–1.75, HPS 2022). COVID-19 vaccination was less likely among participants who reported smoke exposure inside the home (OR = 0.53; 95% CI = 0.31–0.90; 2022), poor water quality (OR = 0.42; 95% CI = 0.21–0.85; 2021), noise from industrial activity/construction (OR = 0.44; 95% CI = 0.19–0.99; 2022), and larger household size (OR = 0.57; 95% CI = 0.46–0.71; HPS 2022). Vaccination was also positively associated with poor indoor air quality (OR = 1.96; 95% CI = 1.02–3.72; 2022) and poor physical housing condition (OR = 2.27; 95% CI = 1.01–5.13; 2022). Certain heating/cooling sources were associated with COVID-19 outcomes.

**Conclusions:**

Our study found poor housing conditions associated with increased COVID-19 burden, which may be driven by systemic disparities in housing, healthcare, and financial access to resources during the COVID-19 pandemic.

## Background

Historic and current policies in the United States (US), such as redlining and exclusionary zoning, have created and perpetuated structural inequities in housing-related environmental conditions [1–4]. Individuals in communities that have been minoritized and marginalized are more likely to be exposed to more lead, dampness, mold, pests, and energy insecurity in the home, as well as more toxic waste and air pollution near the home [5–12]. During the COVID-19 pandemic, these inequities were compounded by additional structural problems including increased housing insecurity and decreased ability to social distance due to household crowding and increased likelihood of having essential worker status [13,14].

While certain housing-related conditions may not directly influence COVID-19 outcomes, other housing-related conditions could affect COVID-19 transmission or severity. The evidence is strongest for household size and household crowding; for example, residence in high-occupancy housing and overcrowding (i.e., more people than rooms) were associated with increased COVID-19 incidence and mortality due to increased likelihood of infection transmission [15–19]. Some evidence also suggests that heating and cooling systems can relate to COVID-19 incidence if the system(s) are associated with effective ventilation [20,21]. Additionally, some housing-related conditions that serve as proxies for socioeconomic status and/or access to health care (e.g., residence in a mobile home, boat, van, or recreational vehicle) are associated with decreased likelihood of having been vaccinated [22]. Finally, other housing-related environmental exposures (e.g., air pollution) are correlated with COVID-19 incidence and can affect susceptibility to severe COVID-19 among those who are infected (e.g., due to associations between the environmental factors and the burden of co-morbidities such as diabetes and asthma that are associated with worse COVID-19 outcomes) [23,24].

Little is known about how other housing-related conditions relate to COVID-19 experiences. Whereas certain housing-related conditions may serve as proxies for known socioeconomic or racial/ethnic disparities, other housing exposures may indirectly affect COVID-19 outcomes independent of other factors. For example, exposure to smoke in the home could exacerbate underlying respiratory or cardiovascular conditions that in turn increase susceptibility to severe COVID-19, once infected. However, to our knowledge, no studies to date have used nationally representative data to quantify the association between a broad set of housing quality indicators and COVID-19 outcomes. Therefore, we used two nationally representative surveys (each administered in 2021 and 2022) to assess the association between various housing-related conditions (e.g., housing quality, household-related environmental conditions, heating/cooling methods) and COVID-19 infection and vaccination outcomes. We used multiple time points from each survey to capture differences in exposure conditions over time, and differences in exposure-outcome associations over time (e.g., possibly related to eligibility for COVID-19 vaccinations and presence of different circulating strains of COVID-19).

## Methods

We used data from two complementary studies with samples designed to be representative of the non-institutionalized adult population in the United States. The primary study, the Tufts Equity in Health, Wealth, and Civic Engagement (TES; n = 1449 in 2021; n = 1831 in 2022), provides rich data on many different household exposures and COVID-19 outcomes. The secondary study, the Household Pulse Survey (HPS; n = 147,380 in 2021; n = 62,826 in 2022), provided a very large data set with which to examine household size and COVID-19 outcomes. Our study was determined to be exempt by the Tufts University Social, Behavioral, and Educational Research Institutional Review Board (protocol STUDY00000428; for analysis of TES data) and Not Human Subjects Research by the Tufts University Health Sciences Institutional Review Board (protocol STUDY00001768; for analysis of HPS data).

### Tufts Equity in Health, Wealth, and Civic Engagement Study (TES): Data Collection and Study Sample

Methods for the TES have been published previously [25]. Briefly, the survey for this study was administered through the Ipsos Public Affairs KnowledgePanel. Initial recruitment into the panel was based on probability-based techniques. Ipsos also uses stratified random sampling methods to maximize geodemographic representativeness of the target US adult population. Once individuals are recruited into the panel, they complete a Core Profile Survey. Subsequently, they become eligible for separate surveys, such as the TES survey. For the present analysis, we used data from 1449 randomly selected KnowledgePanel individuals participating in the second survey wave (April 23-May 3, 2021; 69% completion rate) and 1831 individuals participating in the third survey wave of this study (May 26-June 2, 2022; 66% completion rate). Of these individuals, 1007 participated in both survey waves two (2021) and three (2022). In survey wave three, there were 760 participants who had not participated in a previous survey wave who were selected as part of the study team’s effort to over-sample Hispanic, non-Hispanic African American/Black, and non-Hispanic Asian populations. These participants were randomly selected from among the Hispanic, non-Hispanic African American/Black, and non-Hispanic Asian KnowledgePanel participants.

### TES: Housing Characteristics

Participants self-reported household characteristics including household size (1–2 people/3–4 people/5 or more people), whether they had ever noticed visible water damage, visible mold, musty smells, or moldy smells (yes/no), how often someone (they or others) smoke inside the home (not at all/several days or more frequently in a typical week), the year their housing unit was originally built (asked in 2022 only; 1977 and earlier/1978 and later), whether in the last summer their housing unit was so hot for 24 hours or more that they were uncomfortable (2022 only), whether in the last winter their housing unit was so cold for 24 hours or more that they were uncomfortable (2022 only), and use of candles in the home (2022 only). Additionally, participants stated whether any of the following aspects of their housing negatively affected their health: indoor air quality; indoor temperature; water quality; pests like insects, rodents, or other vermin; physical condition of housing – state of repair; lack of privacy; household members who are exposed to COVID-19 at work or school (2021 only); outdoor air pollution; nighttime noise from road traffic; nighttime noise from aircraft or railways; neighborhood crime or drug activity; lead paint (2022 only), cost of housing (2022 only), noise from industrial activity or construction (2022 only), or none of the above. In 2021 only, participants reported whether they regularly used any of the following heating sources during cold weather months: central gas heating, gas, or oil-fired furnace, or gas or oil-fired boiler; air-source or ground-source heat pump; gas wall heater, space heater, or freestanding combustion heater such as gas or kerosene; electric wall heater, space heater, or freestanding electric heater; wood stove or a gas or wood fireplace; none of the above; or don’t know. Similarly, in 2021, participants reported whether they typically used any of the following cooling sources: central air conditioning; window/wall air conditioning unit(s); fans; open windows; central or room humidifier; evaporative cooling systems; central high efficiency particulate air (HEPA) or electrostatic filter; none of the above; or don’t know. We treated the heating sources question as a proxy for exposure to combustion sources and the cooling sources question as a proxy for ventilation.

### TES: COVID-19 Experiences

We created three dichotomous COVID-19 outcome variables: COVID-19 probable or actual diagnosis, COVID-19 diagnosis (2021 only), and COVID-19 vaccination status.

In the 2021 survey, participants were considered to have had a COVID-19 probable or actual diagnosis if they responded “yes” to either of two questions: *“Have you ever tested positive for COVID-19?”* or *“Although you did not receive a positive test for COVID-19, do you believe you have ever had COVID-19?”* The latter question was included due to difficulty in obtaining COVID-19 diagnostic tests early in the pandemic. Participants were considered not to have had a COVID-19 probable or actual diagnosis if they answered “no” to both questions or if they answered “no” to one question and had missing data for the other question. In 2022, participants were categorized as having had a COVID-19 probable or actual diagnosis based on their response to *“Do you believe that you have had COVID-19?”* (yes/no). Participants who answered “don’t know” were excluded from this analysis.

Participants were considered to have a COVID-19 diagnosis in the 2021 survey if they responded affirmatively to the question *“Have you ever tested positive for COVID-19?”* and were considered to not have had a COVID-19 diagnosis if they responded “no” to this question.

Participants were considered vaccinated or definitely willing to receive a COVID-19 vaccine in the 2021 survey if they either responded “Yes” to *“Have you ever received a COVID-19 vaccine?”* or “Very likely” to *“How likely is it that you will get the vaccine when you are eligible?”* (only asked among respondents who responded “No” or “Unsure” to *“Have you ever received a COVID-19 vaccine?”*). Participants who said “No” or “Unsure” to *“Have you ever received a COVID-19 vaccine?”* and who said they were “Somewhat likely,” “Not sure,” “Somewhat unlikely,” or “Very unlikely” to get a vaccine when they were eligible were considered unvaccinated against COVID-19. Participants were considered vaccinated in the 2022 survey if they reported that they had received one or more doses of a COVID-19 vaccine. Participants who reported that they received zero doses were considered unvaccinated. The 2022 definition differed from the 2021 definition due to the fact that the whole population was not yet eligible to get vaccinated at the time of the 2021 TES survey.

### TES: Covariates

Covariate data for participants in the TES were obtained through self-reported survey responses and through linkage with other data sets. Self-reported covariates included gender (female/male), age (continuous), race/ethnicity (Hispanic/non-Hispanic Black/non-Hispanic White/non-Hispanic other or at least two races), educational attainment (no high school diploma or equivalent/high school graduate or equivalent/some college or Associate’s degree/Bachelor’s degree/Master’s degree or higher), and annual household income (<$25,000/$25,000-$49,999/$50,000-$74,999/$75,000-$99,999/≥$100,000). Based on participants’ self-reported residential ZIP Codes, we used American Community Survey 5-year (2016–2020) estimates to generate median annual household income in 2020 inflation-adjusted dollars (S1901 from the US Census Bureau; <$25,000/$25,000-$49,999/$50,000-$74,999/≥$75,000) [26]. Based on participants’ county of residence, we assessed three additional variables – population density, air pollution exposure, and greenness exposure. Population density was derived using American Community Survey 5-year (2014–2018) estimates in B25010 and B01001 of the US Census Bureau data (< 100/100–499/500–999/1000–1999/≥2000 people per square mile) [27,28]. Air pollution exposure was assessed by the University of Washington in Saint Louis Atmospheric Composition Analysis Group ( https://sites.wustl.edu/acag/datasets/surface-pm2-5/ ) as the 2018 annual average fine particulate matter (PM_2_.5; particles < 2.5 μm in aerodynamic diameter) based on publicly available North American-specific models derived by combining satellite (aerosol optical depth; Terra and Aqua satellites) and ground-monitoring data [29,30]. Greenness exposure was assessed using 16-day composites of normalized difference vegetation index (NDVI; an indicator of photosynthetic activity in plants that ranges from − 1 indicating water to 1 indicating dense green forests) derived from the Moderate Resolution Imaging Spectroradiometer (MODIS) sensor at 250–m × 250–m resolution onboard the Terra satellite (mean across the county of pixel-level non-negative maximum NDVI from April-September 2018) [31]. We dichotomized greenness exposure as high (NDVI > 0.6) or low (NDVI ≤ 0.6) [32].

### Household Pulse Survey (HPS): Data Collection and Study Sample

The HPS is a large, nationally representative household survey conducted by the US Census Bureau in partnership with other federal agencies [33]. Participants were selected using the Census Bureau’s Master Address File January 2021 update (for 2021 data) or January 2022 (for 2022 data), a database with approximately 145 million housing units. Of these housing units, 81% had at least one email address or cell phone number linked in 2021 and 90% had at least one email address or cell phone number linked in 2022.[34] The present analysis uses data from two merged waves of Phase 3.1 (2021) and one wave from Phase 3.5 (2022). Details of the systematic sampling strategy and weighting procedures are provided elsewhere [34,35]. Briefly, we used data from 68,913 participants who responded to the Qualtrics survey between April 14 and April 26, 2021 (wave one; 6.6% response rate), 78,467 participants who responded to the survey between April 28 and May 10, 2021 (wave two; 7.4% response rate), and 62,826 participants who responded to the survey between June 1 and June 13, 2022 (6.2% response rate) [34,35]. These survey waves were selected since they were the closest temporally to the data collection time periods for the TES. We used the HPS to validate findings from the TES, as the HPS has a much larger sample.

### HPS: Housing Characteristics

Participants in the HPS were asked to indicate a number for: *“How many total people – adults and children – currently live in your household, including yourself?”* We categorized this variable with the levels of 1–2 people, 3–4 people, and five or more people.

### HPS: COVID-19 Experiences

We used two COVID-19 outcomes – COVID-19 diagnosis and COVID-19 vaccination status.

For COVID-19 diagnosis in 2021, participants were asked: *“Has a doctor or other health care provider ever told you that you have COVID-19?”* For the COVID-19 diagnosis in 2022, participants were asked: *“Have you ever tested (using a rapid point-of-care test, self-test, or laboratory test) positive for COVID-19 or been told by a doctor or other health care provider that you have or had COVID-19?”* We included participants who responded “yes” and “no” in these analyses.

For COVID-19 vaccination status in 2021, participants who indicated “yes” to *“Have you received a COVID-19 vaccine?” or “definitely get a vaccine”* to *“Once a vaccine to prevent COVID-19 is available to you, would you…”* were considered vaccinated or definitely willing to vaccinate. Participants who answered “no” to *“Have you received a COVID-19 vaccine?”* and any of “probably get a vaccine,” “be unsure about getting a vaccine,” “probably NOT get a vaccine,” or “definitely NOT get a vaccine” to *“Once a vaccine to prevent COVID-19 is available to you, would you…”* were considered unvaccinated. For COVID-19 vaccination status in 2022, we categorized people based on their response to *“Have you received a COVID-19 vaccine?”* (yes/no).

### HPS: Covariates

Self-reported covariate data for participants in the HPS included gender (female/male), age (continuous), race/ethnicity (Hispanic/non-Hispanic Black/non-Hispanic White/non-Hispanic other or at least two races), educational attainment (no high school diploma or equivalent/high school graduate or equivalent/some college or Associate’s degree/Bachelor’s degree/above a Bachelor’s degree), annual household income (<$25000/$25,000-$49,999/$50,000-$74,999/$75,000-$99,999/≥$100,000), and residence in a metropolitan statistical area (yes/no for any of: Atlanta-Sandy Springs-Alpharetta (GA), Boston-Cambridge-Newton (MA-NH), Chicago-Naperville-Elgin (IL-IN), Dallas-Fort Worth-Arlington (TX), Detroit-Warren-Dearborn (MI), Houston-The Woodlands-Sugar Land (TX), Los Angeles-Long Beach-Anaheim (CA), Miami-Fort Lauderdale-Pompano Beach (FL), New York-Newark-Jersey City (NY-NJ), Philadelphia-Camden-Wilmington (PA-NJ-DE-MD), Phoenix-Mesa-Chandler (AZ), Riverside-San Bernardino-Ontario (CA), San Francisco-Oakland-Berkeley (CA), Seattle-Tacoma-Bellevue (WA), or Washington-Arlington-Alexandria (DC-VA-MD-WV)).

### Statistical Analyses and Conceptual Model

All analyses were conducted in Stata/SE v17 and employed survey weighting to maximize the representativeness of the sample. All analyses for the two studies (TES and HPS) and the two years (2021 and 2022) were conducted separately to capture differences in housing conditions over time, and differences in exposure-outcome associations over time. For each study, we first examined descriptive characteristics of the samples. We calculated weighted counts and proportions for all categorical variables, and we calculated means and 95% confidence intervals (95% CIs) for all continuous variables. Next, for the TES, we examined correlations between heating sources and cooling sources. We created new exposure variables for pairs of heating and cooling sources that were significantly associated with each other (Pearson’s chi-squared p-value < 0.05) and that had at least 25 people (based on weighted counts) who reported that they used both heating and cooling source in the pair. Then, we estimated logistic regression models for the cross-sectional associations between each exposure variable and each outcome variable (separate models).

To estimate associations between each household exposure (other than household size) and each outcome using data from the TES, we fit unadjusted models and two sets of adjusted models. The primary models were adjusted for covariates determined using an evidence-based directed acyclic graph (DAG) and included age, race/ethnicity, annual household income, county-level population density, county-level annual air pollution, and county-level greenness (Supplemental Fig. 1). As a sensitivity analysis, the second set of models were additionally adjusted for gender, educational attainment, and ZIP Code level median household income since these variables were identified through the DAG development process as related to the exposure and outcome (though not as part of the minimally sufficient adjustment set). Please refer to the supplement for details of the DAG development, along with the DAG code and justification for each arrow.

To estimate associations between household size and the outcomes, we fit unadjusted and adjusted models (separate models for each outcome and study). The set of covariates in the adjustment set was determined based on the DAG (see Supplemental Text and Supplemental Fig. 1 for details). For models using the TES data, covariates included age, gender, race/ethnicity, educational attainment, annual household income, ZIP Code level median household income, and county-level population density. The adjustment set for the HPS was similar, except that we included residence in a metropolitan statistical area instead of county-level population density and we did not include ZIP Code level median household income (based on lack of available ZIP Code data).

## Results

Participants had similar demographic characteristics across the two studies and two time periods (Table 1). In each, 51–52% of participants were female, the average age was 48–50 years, 61–63% of participants were non-Hispanic White, and 31–35% had earned a Bachelor’s degree or higher. Participants in the TES were more likely to earn $75,000 or more (52.4% versus 44.4% in 2021), more likely to live in more densely populated areas (39.0% resided in a county with 1000 or more people/square mile versus 33.5% of HPS participants resided in a metropolitan statistical area in 2021), and more likely to reside in households with 1–2 people (54.3% versus 39.6% in 2021). Participants in the TES were less likely to have a probable or actual COVID-19 case in 2021 (10.4% versus 14.2%) and were less likely to vaccinate against COVID-19 (66.2% versus 78.1% in 2021; 81.6% versus 84.4% in 2022).

### Housing conditions

In the TES, most of the adverse housing conditions were relatively uncommon with 64% reporting that none of the aspects of their housing negatively affected their health in 2021 and 61% reporting the same in 2022 (Table 1). However, 11% reported that noise negatively affected their health (2021 and 2022), 15% (in 2021) and 17% (in 2022) reported that pests or the physical condition of their housing negatively affected their health, and approximately one-third (29% in 2021, 35% in 2022) reported that they had noticed visible water damage, visible mold, musty smells, or moldy smells in their current home. Additionally, about one in five participants (20% in 2021 and 18% in 2022) reported that people smoked inside their home or that indoor air quality negatively affected their health. In general, there were strong positive correlations across the reported housing conditions (Supplemental Fig. 2).

In the primary models, having visible water damage/mold or musty/moldy smells was associated with increased odds of having a probable or actual COVID-19 diagnosis by 2022 (adjusted odds ratio [aOR] = 1.50; 95% CI = 1.10, 2.03) whereas the presence of pests was associated with lower odds of having a probable or actual COVID-19 diagnosis by 2022 (aOR = 0.60; 95% CI = 0.39, 0.95). Having a house in poor physical condition was associated with increased odds of having been diagnosed with COVID-19 by 2021 (aOR = 2.32; 95% CI = 1.17, 4.59). Vaccination was significantly less likely for people reporting smoking inside the home in 2021 (aOR = 0.40; 95% CI = 0.25, 0.63) and 2022 (aOR = 0.53; 95% CI = 0.31, 0.90), poor water quality affecting their health in 2021 (aOR = 0.42; 95% CI = 0.21, 0.85), and noise from industrial activity or construction affecting their health in 2022 (aOR = 0.44; 95% CI = 0.19, 0.99). In contrast, people were more likely to be vaccinated in 2022 if they reported poor indoor air quality affecting their health (aOR = 1.96; 95% CI = 1.02, 3.72) or poor physical condition of their housing affecting their health (aOR = 2.27; 95% CI = 1.01, 5.13). None of the other housing conditions were significantly associated with any of the COVID-19 outcomes in the primary models (Table 2). Most of the trends observed in the primary models were also observed in the unadjusted and alternatively adjusted models (Supplemental Table 1).

### Heating and cooling sources (2021 data only)

Central heating and central air conditioning were the most common heating and cooling sources for TES participants (57.9% and 70.2%, respectively), with 44.5% of participants having both in 2021. Additionally, there were 12 other combinations of heating and cooling sources that were significantly correlated and had at least 25 people (based on weighted counts) who reported that they used both sources in the pair (Supplemental Table 2). People who did not have any heating source or any cooling source tended to live in the South Atlantic (34.4% for heating; 24.5% for cooling) or Pacific (23.6% for heating; 49.5% for cooling) regions of the US.

In the primary 2021 models, having window/wall air conditioning was significantly associated with increased odds of having a probable or actual COVID-19 diagnosis (aOR = 1.57; 95% CI = 1.06, 2.32). None of the other individual heating or cooling sources was significantly associated with any of the COVID-19 outcomes (Table 3 for primary results; unadjusted and sensitivity analyses in Supplemental Table 3). However, three combinations of heating/cooling sources were significantly associated with lower odds of having had a COVID-19 diagnosis (heat pump with open windows; space heater (combustion or electric) with open windows; wood stove or gas/wood fireplace with fans) and one combination of a heating/cooling source (central gas, or gas/oil furnace or boiler with window/wall air conditioning) was significantly associated with a higher likelihood of having a probable or actual COVID-19 diagnosis (Table 3). Additionally adjusting the primary models for geographic region did not substantively change the results, except that the inverse association between using a space heater (combustion or electric) with open windows for cooling and having a probable or actual COVID-19 diagnosis became slightly stronger (aOR = 0.32, 95% CI = 0.11, 0.94). Similarly, adjusting the primary models for home ownership did not substantively change the results except that the associations with vaccination were stronger for the use of heat pumps (aOR = 1.76; 95% CI = 1.00, 3.11) and window/wall air conditioning (aOR = 0.66; 95% CI = 0.45, 0.96).

### Household size

Household size was associated with COVID-19 outcomes in both the TES and HPS, though likely due to statistical power considerations, the trends were more robust in the HPS (Table 4). For example, having five or more people in the household (versus 1–2 people) was significantly associated with double the odds of having a COVID-19 diagnosis (crude OR = 2.00; 95% CI = 1.17, 3.44) in the 2021 TES unadjusted model but the association was attenuated and non-significant in the adjusted model (aOR = 1.71; 95% CI = 0.93, 3.17). In contrast, while the point estimates in the HPS were similar to those in the TES, having five or more people in the household (versus 1–2 people) was significantly associated with increased odds of having had COVID-19 in both 2021 and 2022 in the HPS. Additionally, five or more people in the household (versus 1–2 people) was significantly associated with lower odds of vaccination in the HPS in both 2021 (aOR = 0.52; 95% CI = 0.47, 0.57) and 2022 (aOR = 0.57; 95% CI = 0.46, 0.71).

## Discussion

Our study was the first to quantitatively examine associations between a variety of housing-related conditions and COVID-19 outcomes using data from two nationally representative studies and adjusting for a set of covariates informed by an evidence-based directed acyclic graph. Larger household size as well as reports of adverse housing conditions (e.g., poor physical condition of housing or visible water damage/mold or musty/moldy smells) were associated with increased likelihood of having had COVID-19. Similarly, individuals were less likely to be vaccinated if they reported that people smoked inside their home, or if they reported that water quality or noise from industrial activity or construction adversely affected their health. Additionally, our findings provide a unique perspective on housing conditions among a nationally representative sample. For example, approximately one-third of people had noticed visible water damage, visible mold, musty smells, or moldy smells in their home (29% in 2021, 35% in 2022). Our study adds to the literature supporting investment in structural public health and public policy measures that promote equitable access to safe and healthy housing. These measures would likely help advance future pandemic preparedness efforts by mitigating indirect and direct pathways relating housing exposures and infectious disease outcomes.

Most previous literature relating housing conditions to COVID-19 outcomes focused on household size as a proxy for crowding or number of out-of-home social contacts [15–19]. In particular, increasing household size generally increases the total number of out-of-home social contacts and opportunities for transmission both in the community and within the home [36,37]. Concordant with the conclusions in those studies, we observed that larger household size was associated with increased likelihood of having had a COVID-19 diagnosis and decreased likelihood of vaccination. This finding was particularly robust in our analysis with the HPS data – a large survey rigorously conducted by the U.S. Census Bureau. It is also concordant with previous HPS analyses of the relationship between household size and vaccination status [38]. It suggests that factors associated with household size – such as socioeconomic status, age and family structure, household activities, and cultural norms – may all affect disparities in COVID-19 burden.

Similarly, to the extent that different heating and cooling methods relate to differential ventilation quality within the home, there can be direct relationships between heating and cooling methods and COVID-19 outcomes [21,39]. We observed some evidence that having window/wall air conditioning units (but not central air conditioning) was associated with increased likelihood of having a probable or actual COVID-19 diagnosis. This observation could be explained by differences in amount of air circulation and air filtration effectiveness of different units; a hypothesis supported by our observation that COVID-19 diagnosis was 91% less likely among participants who used open windows as a cooling system in combination with heat pumps as a heating system (heat pumps use electricity to transfer thermal energy). Additionally, it seems unlikely that residual confounding by socioeconomic status completely explains our associations, especially since adjusting for home ownership, income, and educational attainment did not substantively change most of the results. Although participants with window or wall air conditioning units were significantly more likely to have lower levels of educational attainment and lower incomes, people who used window or wall air conditioning units in combination with central gas or gas/oil furnace or boilers were also significantly more likely to have had COVID-19 (as in the primary finding) but these heating sources were more commonly used among people with higher educational attainment and income. Future studies with larger sample sizes could further explore how heating and cooling combinations affect COVID-19 transmission patterns, and how these relationships are affected by regional COVID-19 vaccination trends or other local infection control policies (though notably, we did not observe substantial differences when we adjusted for geographic region). Future studies should also directly measure effectiveness of heating, ventilation, filtration system, and air conditioning systems – accounting for frequency and patterns of use – on COVID-19 transmission.

Most of the other housing-related conditions we examined are not expected to have a direct relationship with COVID-19 diagnosis or vaccination; rather, the housing conditions may have an indirect relationship with COVID-19 (e.g., air pollution exposure is associated with increased likelihood of health outcomes such as diabetes that increase the likelihood for COVID-19 mortality) [23,24] or serve as proxies for other structural disparities (e.g., socioeconomic status, ability to socially isolate, access to care) that affect likelihood of developing COVID-19 or accessing vaccinations [40–45]. The latter hypothesis was supported by our data; for example, even after adjustment for annual household income and other covariates, some of the strongest associations between housing quality conditions and COVID-19 outcomes were observed for variables such as perception that poor physical condition of housing had a negative impact on the participant’s health. These results provide further justification that it is worthwhile to invest in structural equity – including in programs and policies that promote access to safe, affordable, and healthy housing.

Underlying the need for such an investment in housing quality, we found that over one-third of participants reported that at least one aspect of their housing negatively affected their health. The proportion slightly increased from 2021 (36%) to 2022 (39%). Pests, poor indoor air quality, and noise (from road traffic, aircraft, railways, industrial activity, or construction) were all common concerns. Similarly, approximately one-third of participants reported having visible water damage, visible mold, moldy smells, or musty smells in their home. The ability to examine housing quality in such detail using data from a nationally representative sample was a major strength of our analysis.

Another strength of our analysis was that we could examine associations between housing characteristics and COVID-19 outcomes in different phases of the pandemic. In general, the magnitude and direction of the associations were similar across the two survey years. Among the few exceptions, the perception of poor physical condition of housing was positively associated with COVID-19 diagnosis in 2021 (but not 2022) and with vaccination status in 2022 (but not 2021). These results in the TES data perhaps suggests that COVID-19 health outcomes within the first year of the pandemic increased likelihood of vaccination by 2022. However, we did not directly assess whether having had a prior COVID-19 infection was associated with vaccination status in this analysis, and our previous work with the HPS indicated that having had a previous COVID-19 infection was negatively associated with vaccination status [38,42]. An alternative explanation is that the way people self-reported their perception of whether the physical condition of their housing affected their health changed between 2021 and 2022, perhaps due to differences in likelihood of working from home and spending more time at home.

Our study had several other strengths. We had two complementary nationally representative surveys – one with a very large sample size and one with rich characterization of housing quality and multiple waves of survey data on the same participants. Surveys were fielded in English and Spanish. Additionally, we were able to adjust for important confounding variables identified through a rigorous, evidence-based approach. Relatedly, the directed acyclic graph we generated could be useful for researchers in other contexts. Given the similarities in the demographic characteristics between the two studies and our survey weighting methodology, it seems unlikely that selection bias was a major limitation.

Our study also had several limitations. It is possible that there was social desirability bias in how participants self-reported exposures and outcomes. If there were, we likely underestimated true associations as it might be expected that people were less likely to report both the extent of adverse housing conditions and adverse COVID-19 outcomes. There could be exposure or outcome misclassification for reasons other than social desirability. For example, not all people may be aware of their housing characteristics and exposures; for example, people who rent may be less likely to know about the heating and cooling systems used in their homes. We also did not account for frequency or intensity of housing exposures. Additionally, people may have incorrectly reported their COVID-19 diagnoses – especially given the difficulty in obtaining COVID-19 diagnostic tests early in the pandemic; we included the ‘actual or probable diagnosis’ variable to address this possibility and the related concern about differential outcome misclassification if people who had less access to tests also had more adverse housing exposures. Furthermore, we could not account for temporal trends and do not claim that the trends we observed were necessarily causal; future work using longitudinal data would be needed to assess these types of relationships. Due to sample size considerations (especially in the TES), we could not disaggregate certain races/ethnicities. Finally, in the HPS, we did not have data on certain covariates (i.e., ZIP Code level median household income and county-level population density) so we used alternative measures (i.e., residence in a metropolitan statistical area).

## Conclusion

We identified a substantial burden of adverse housing conditions using data in two survey years from each of two nationally representative samples. Housing conditions, such as household size and physical condition of housing, were associated with COVID-19 diagnosis and vaccination. Overall, our study suggests that structural equity issues associated with poor housing conditions are also associated with increased COVID-19 burden, even after adjusting for traditional social determinants of health such as income and race/ethnicity. Addressing disparities in housing conditions would advance public health goals.

## Figures and Tables

**Table 1:** Descriptive characteristics of the two study populations during each survey year

Characteristic	Tufts Equity in Health, Wealth, and Civic Engagement Study weighted mean or % (95% confidence interval)		Household Pulse Survey weighted mean or % (95% confidence interval)^1^	
	2021 N = 1449	2022 N = 1831	2021 N = 147,380	2022 N = 62,826
**Female**	51.6 (48.4, 54.8)	51.1 (47.7, 54.4)	51.6(51.6, 51.6)	51.1 (50.9, 51.3)
**Age** (years)	48.4 (47.2, 49.6)	49.5 (48.3, 50.7)	48.5 (48.4, 48.6)	49.0 (48.9, 49.1)
**Race/ethnicily**				
non-Hispanic White	62.9 (60.0, 65.8)	61.1 (58.1, 64.0)	62.3 (62.2, 62.4)	62.1 (61.9, 62.2)
non-Hispanic Black	11.8 (10.4, 13.4)	12.5(11.1, 14.1)	11.5(11.4, 11.6)	11.4 (11.2, 11.5)
non-Hispanic other or multi-race	8.7 (6.7,11.1)	8.7 (7.4,10.2)	9.0 (8.9, 9.1)	9.2 (9.1,9.4)
Hispanic	16.5(14.7, 18.5)	17.7 (15.8, 19.7)	17.2 (17.1, 17.3)	17.3 (17.1, 17.5)
**Educational attainment**				
No high school diploma	11.2 (9.3,13.5)	9.5 (7.7,11.6)	8.4 (8.1, 8.6)	7.6 (7.2, 8.1)
High school graduate or equivalent	27.4 (24.6, 30.3)	28.4 (25.5, 31.4)	30.6 (30.3, 30.8)	31.2 (30.7, 31.6)
Some college or Associate's degree	29.9 (27.0, 33.0)	27.0 (24.0, 30.1)	30.2 (30.2, 30.2)	30.4 (30.3, 30.4)
Bachelor’s degree	18.4 (16.1, 21.0)	19.0 (16.6, 21.7)	17.2 (17.1, 17.4)	17.2 (16.9, 17.4)
Master’s degree or higher	13.2 (11.2, 15.4)	16.1 (13.7, 18.9)	13.6(13.5, 13.8)	13.7 (13.4, 14.0)
**Annual household income**				
Less than $25,000	12.6 (10.6, 14.9)	12.6 (10.4, 15.1)	14.4 (14.0, 14.9)	14.5(13.9, 15.1)
$25,000 to $49,999	17.6 (15.3, 20.1)	16.7 (14.4, 19.3)	23.7 (23.2, 24.2)	23.4 (22.7, 24.2)
$50,000 to $74,999	17.4 (15.1, 20.0)	15.7 (13.3, 18.3)	17.5(17.1, 17.9)	17.2 (16.5, 17.9)
$75,000 to $99,999	14.0 (11.9, 16.4)	13.5(11.5, 15.7)	13.5(13.1, 14.0)	12.9 (12.4, 13.5)
$100,000 or more	38.4 (35.3, 41.6)	41.5(38.2, 44.9)	30.9 (30.5, 31.3)	31.9 (31.2, 32.6)
**Median household income** (ZIP-code)				
Less than $25,000	0.4 (0.2, 0.8)	0.4 (0.2, 0.8)		
$25,000 to $49,999	22.2 (19.7, 24.8)	23.0 (20.4, 25.8)		
$50,000 to $74,999	40.0 (36.8, 43.2)	38.7 (35.5, 42.1)		
$75,000 or more	37.5 (34.4, 40.7)	37.9 (34.6, 41.2)		
**Annual average PM_2.5_** (μg/m^3^; county)	7.5 (7.4, 7.6)	7.3 (7.2, 7.4)		
**High greenness^2^** (county)	78.7 (76.1, 81.1)	78.0 (75.3, 80.4)		
**Population density** (county)				
Fewer than 100 people/square mile	16.7 (14.4, 19.3)	17.1 (14.6, 20.0)		
100–499 people/square mile	30.7 (27.7, 33.9)	31.9 (28.6, 35.3)		
500–999 people/square mile	13.6 (11.6, 15.9)	12.2 (10.3, 14.3)		
1000–1999 people/square mile	17.7 (15.4, 20.2)	18.3 (16.0, 20.7)		
2000 or more people/square mile	21.3 (18.8, 24.0)	20.6 (18.2, 23.2)		
**Residence in a metropolitan area^3^**			33.5 (32.8, 34.1)	32.5(31.8, 33.2)
**Exposure: Household size**				
1 person	18.5(16.1, 21.1)	19.0 (16.5, 21.8)	7.8 (7.5, 8.1)	9.1 (8.7, 9.5)
2 people	35.8 (32.8, 38.9)	38.4 (35.2, 41.7)	31.8(31.3, 32.3)	32.3 (31.6, 33.0)
3 people	19.0 (16.6, 21.6)	14.9 (12.7, 17.4)	19.6(19.3, 20.0)	18.9 (18.2, 19.7)
4 people	15.2 (12.8, 17.9)	15.5(13.0, 18.3)	19.4 (19.0, 19.9)	19.1 (18.5, 19.7)
5 or more people	11.6 (9.6,13.8)	12.3 (10.3, 14.7)	21.3 (20.6, 22.0)	20.6 (19.7, 21.5)
**Exposure: Visible water damage or mold, or musty or moldy smell**	29.4 (26.5, 32.4)	34.5(31.3, 37.7)		
**Exposure: Smoking inside the home**	10.0 (8.2,12.2)	9.4 (7.6,11.6)		
**Exposure: Uncomfortable indoor temperature^4^**		22.9 (20.3, 25.7)		
**Exposure: Use of candles in home**		59.7 (56.3, 63.0)		
**Exposure: Lead^5^**		43.9 (40.6, 47.3)		
**Exposures: Housing conditions with negative impact on health**				
Poor outdoor air quality	9.5 (7.9,11.5)	8.1 (6.5,10.0)		
Poor indoor air quality	11.4 (9.4,13.7)	10.1 (8.3,12.2)		
Poor indoor temperature	6.0 (4.7, 7.7)	7.1 (5.7, 8.7)		
Poor water quality	5.7 (4.4, 7.4)	6.5 (5.3, 8.0)		
Poor physical condition	7.5 (5.9, 9.5)	8.1 (6.3,10.2)		
Lead		2.1 (1.4, 3.1)		
Presence of pests	11.2 (9.2,13.6)	14.1 (11.9, 16.6)		
Nighttime noise from road traffic	8.4 (6.6,10.6)	7.9 (6.4, 9.6)		
Nighttime noise from aircraft or railways	4.5 (3.4, 6.1)	4.1 (2.9, 5.8)		
Noise from industrial activity or construction		2.6 (1.8, 3.8)		
Neighborhood crime or drug activity	6.3 (4.9, 8.1)	7.7 (6.3, 9.4)		
Lack of privacy	4.2 (3.1, 5.7)	5.1 (4.0, 6.6)		
Household member exposed to COVID- 19	2.9 (2.1,4.2)			
Housing cost		12.3 (10.2, 14.8)		
**Exposures: Heating source**				
Central gas, or gas/oil furnace or boiler	57.9 (54.6, 61.0)			
Heat pump	9.5 (7.8,11.5)			
Space heater (combustion or electric)	8.1 (6.6,10.0)			
Wood stove or gas or wood fireplace	11.0 (9.1,13.2)			
**Exposures: Cooling source**				
Central air conditioning	70.2 (67.1, 73.0)			
Window/wall air conditioning	19.8 (17.3, 22.5)			
Fans	35.7 (32.7, 38.9)			
Open windows	31.5(28.6, 34.6)			
Evaporative cooling, humidifier, or HEPA or electrostatic filter	3.2 (2.2, 4.4)			
**COVID-19 outcomes**				
Probable or actual diagnosis	21.1 (18.6, 23.8)	41.3 (37.9, 44.9)		
Diagnosis	10.4 (8.7,12.4)		14.2 (13.7, 14.6)	40.3 (39.5, 41.1)
Vaccinated or definitely willing to vaccinate	66.2 (63.0, 69.3)	81.6 (78.6, 84.2)	78.1 (77.7, 78.6)	84.4 (83.8, 84.9)

1 Weighted to the US population. Total sample size = 147,380 people.

2 High greenness = normalized difference vegetation index exposure value > 0.6.

3 Metropolitan areas = residence in one of 15 designated metropolitan statistical areas.

4 Uncomfortable indoor temperature = housing unit uncomfortably hot in summer for at least 24 hours, uncomfortably cold in winter for at least 24 hours, or indoor temperature perceived as negatively affecting health

5 Lead = housing unit built in 1977 or earlier or reported that lead paint in housing negatively affects health

**Table 2:** Associations between self-reported household exposures and COVID-19 outcomes in the Tufts Equity in Health, Wealth, and Civic Engagement Study: United States, 2021 and 2022

Exposure	Probable or actual COVID-19 diagnosis 2021 - aOR (95% Cl)^1^ 2022 - aOR (95% Cl)	COVID-19 diagnosis 2021 - aOR (95% Cl)	Vaccinated/definitely willing to vaccinate for COVID-19 2021 - aOR (95% Cl) 2022 - aOR (95% Cl)
Visible water damage or mold, or musty or moldy smell	1.34 (0.95,1.89) 1.50 (1.10, 2.03)*	1.14(0.74, 1.75)	0.74 (0.53,1.03) 0.94 (0.63,1.40)
Smoking inside the home	0.61 (0.31,1.19) 1.20 (0.69, 2.10)	0.62 (0.22, 1.73)	0.40 (0.25, 0.63)* 0.53 (0.31,0.90)*
Uncomfortable indoor temperature	0.87 (0.60,1.27)		1.04 (0.64,1.67)
Use of candles in the home	0.75(0.54,1.02)		0.87 (0.58,1.32)
Lead	1.20 (0.87,1.65)		1.04 (0.69,1.58)
Negative impact: Poor outdoor air quality	1.14(0.70,1.88) 0.64 (0.36,1.13)	0.78 (0.40, 1.53)	0.83 (0.51,1.35) 2.13(0.87, 5.21)
Negative impact: Poor indoor air quality	1.47 (0.90, 2.40) 1.04 (0.64,1.67)	1.09 (0.59, 1.99)	0.78 (0.47,1.30) 1.96 (1.02,3.75)*
Negative impact: Poor indoor temperature	1.22 (0.68, 2.16) 0.80(0.49,1.31)	1.52 (0.76, 3.04)	1.13 (0.61,2.12) 1.18(0.62, 2.25)
Negative impact: Poor water quality	1.36(0.76, 2.43) 1.18(0.70,1.98)	0.70 (0.28, 1.76)	0.42 (0.21,0.85)* 0.71 (0.38,1.33)
Negative impact: Poor physical condition	1.75(0.99,3.10) 0.76(0.43,1.35)	2.32 (1.17, 4.59)*	0.71 (0.37,1.34) 2.27 (1.01,5.13)*
Negative impact: Lead	1.36(0.52,3.56)		1.27 (0.30, 5.41)
Negative impact: Presence of pests	1.42 (0.86, 2.35) 0.60 (0.39, 0.95)*	0.99 (0.54, 1.81)	0.93 (0.56,1.52) 1.08 (0.59,1.97)
Negative impact: Nighttime noise from road traffic	0.93 (0.50,1.71) 1.19(0.74,1.91)	0.86 (0.40, 1.84)	1.18(0.64, 2.19) 0.85(0.46,1.59)
Negative impact: Nighttime noise from aircraft or railways	1.23 (0.59, 2.58) 0.92 (0.37, 2.25)	0.56 (0.17, 1.82)	0.59 (0.28,1.25) 1.30 (0.46,3.63)
Negative impact: Noise from industrial activity or construction	0.52 (0.21,1.28)		0.44 (0.19,0.99)*
Negative impact: Neighborhood crime or drug activity	1.19(0.66, 2.13) 1.34 (0.84, 2.12)	1.25 (0.65, 2.38)	1.01 (0.51,2.00) 0.91 (0.49,1.71)
Negative impact: Lack of privacy	1.47 (0.73, 2.96) 1.02 (0.55,1.90)	0.69 (0.21, 2.27)	0.51 (0.24,1.11) 1.12(0.51,2.46)
Negative impact: Household member exposed to COVID-19	1.63 (0.74,3.60)	2.22 (0.94, 5.29)	1.56 (0.53,4.62)
Negative impact: Housing cost	0.89 (0.54,1.48)		1.12(0.61,2.04)

* : p < 0.05

1 OR: adjusted odds ratio; Cl = confidence interval; p = value; Adjusted for age, race/ethnicity, annual household income, county-level population density, county-level annual air pollution, and county-level greenness. Covariates determined using the evidence-based directed acyclic graph.

**Table 3:** Associations between self-reported heating and cooling sources and COVID-19 outcomes in the second wave(2021) of the Tufts Equity in Health, Wealth, and Civic Engagement Study

Exposure	Probable or actual COVID-19 diagnosis aOR (95% Cl)^1^	COVID-19 diagnosis aOR (95% Cl)	Vaccinated/definitely willing to vaccinate for COVID-19 aOR (95% CI)
Heating source: Central gas, or gas/oil furnace or boiler	1.15 (0.82, 1.60)	1.20 (0.77, 1.89)	1.21 (0.89,1.64)
Heating source: Heat pump	0.93 (0.53, 1.66)	0.86 (0.38, 1.98)	1.56 (0.89, 2.73)
Heating source: Space heater (combustion or electric)	0.91 (0.51, 1.60)	1.43 (0.72, 2.82)	0.77 (0.44,1.36)
Heating source: Wood stove or gas/wood fireplace	1.45 (0.89, 2.35)	0.81 (0.40, 1.63)	0.70 (0.44,1.13)
Heating source: None	0.92 (0.58, 1.47)	0.99 (0.55, 1.79)	0.93 (0.59,1.47)
Cooling source: Central air conditioning	0.84 (0.59, 1.20)	0.81 (0.52, 1.24)	1.31 (0.95,1.83)
Cooling source: Window/wall air conditioning	1.57 (1.06, 2.32)*	1.53 (0.94, 2.48)	0.72 (0.50,1.03)
Cooling source: Fans	0.90 (0.65, 1.26)	0.75 (0.48, 1.16)	1.00 (0.73,1.37)
Cooling source: Open windows	0.87 (0.61, 1.23)	0.71 (0.45, 1.13)	1.25 (0.90,1.73)
Cooling source: Evaporative cooling, humidifier, or HEPA or electrostatic filter	0.99 (0.45, 2.20)	1.34 (0.49, 3.67)	0.78 (0.31,1.97)
Cooling source: None	0.48 (0.05, 4.83)	^2^	0.52 (0.14,1.97)
Heating/cooling combination: Central gas, orgas/oil furnace or boiler with central air conditioning	0.87 (0.63, 1.21)	0.84 (0.54, 1.28)	1.25 (0.92,1.69)
Heating/cooling combination: Central gas, orgas/oil furnace or boiler with window/wall air conditioning	2.13 (1.29, 3.50)*	1.64 (0.91 2.94)	0.73 (0.43,1.22)
Heating/cooling combination: Heat pump with central air conditioning	0.99 (0.54, 1.80)	0.92 (0.39, 2.16)	1.56 (0.87, 2.79)
Heating/cooling combination: Heat pump with fans	0.86 (0.27, 2.74)	^2^	1.71 (0.58, 5.05)
Heating/cooling combination: Heat pump with open windows	0.34 (0.09, 1.38)	0.09 (0.01 0.67)*	1.47 (0.34, 6.27)
Heating/cooling combination: Space heater (combustion or electric) with central air conditioning	0.88 (0.36, 2.14)	1.86 (0.71 4.88)	0.95 (0.34, 2.68)
Heating/cooling combination: Space heater (combustion or electric) with window/wall air conditioning	0.73 (0.28, 1.91)	1.12 (0.35, 3.57)	0.73 (0.38,1.40)
Heating/cooling combination: Space heater (combustion or electric) with fans	0.96 (0.43, 2.17)	0.88 (0.27, 2.87)	0.79 (0.34,1.84)
Heating/cooling combination: Space heater (combustion or electric) with open windows	0.35(0.12, 1.01)	0.15 (0.03, 0.69)*	1.09 (0.39,3.03)
Heating/cooling combination: Space heater (combustion or electric) with evaporative cooling, humidifier, or HEPA or electrostatic filter	1.16 (0.42, 3.19)	1.76 (0.53, 5.86)	0.56 (0.16,1.93)
Heating/cooling combination: Wood stove or gas/wood fireplace with fans	0.77 (0.41, 1.46)	0.35 (0.13, 0.94)*	0.63 (0.33,1.23)
Heating/cooling combination: Wood stove or gas/wood fireplace with open windows	0.99 (0.51, 1.89)	0.60 (0.22, 1.63)	0.76 (0.38,1.52)
Heating/cooling combination: No heating with central air conditioning	0.97 (0.56, 1.67)	0.99 (0.51 1.92)	0.86 (0.50,1.47)

* : p < 0.05

1 OR: adjusted odds ratio; Cl = confidence interval; p = value; Adjusted for age, race/ethnicity, annual household income, county-level population density, county-level annual air pollution, and county-level greenness. Covariates determined using the evidence-based directed acyclic graph.

2 Not applicable. No people with exposure and outcome.

**Table 4:** Associations between household size and COVID-19 outcomes

Dataset	Probable or actual COVID-19 diagnosis		COVID-19 diagnosis		Vaccinated/definitely willing to vaccinate for COVID-19	
	Unadjusted OR (95% CI; p)^1^	Adjusted OR (95% CI; p)^2^	Unadjusted OR (95% CI; p)	Adjusted OR (95% CI; p)	Unadjusted OR (95% Cl; p)	Adjusted OR (95% Cl; p)
**Tufts Equity in Health, Wealth, ana Civic Engagement Study**						
**2021**						
1–2 people	reference	reference	reference	reference	reference	reference
3–4 people	1.51 (1.07, 2.15)*	1.37 (0.93 2.01)	1.39 (0.88, 2.19)	1.18 (0.73 1.92)	0.67 (0.49, 0.91)*	0.76 (0.53 1.09)
5 or more people	1.79 (1.13, 2.83)*	1.58 (0.95, 2.62)	2.00(1.17, 3.44)*	1.71 (0.93 3.17)	0.72 (0.46, 1.13)	1.05 (0.61 1.78)
**2022**						
1 –2 people	reference	reference			reference	reference
3–4 people	1.19 (0.86, 1.66)	0.99 (0.70, 1.41)			0.65 (0.42, 0.99)*	0.62 (0.39 0.99)*
5 or more people	1.61 (1.04, 2.50)*	1.36 (0.84, 2.19)			0.95 (0.57, 1.61)	1.07 (0.55, 2.09)
**Household Pulse Survey**						
**2021**						
1–2 people			reference	reference	reference	reference
3–4 people			1.46 (1.36, 1.57)*	1.28 (1.17, 1.40)*	0.63 (0.60, 0.67)*	0.77 (0.71 0.82)*
5 or more people			2.16(1.95, 2.38)*	1.79 (1.58, 2.03)*	0.38 (0.36, 0.41)*	0.52 (0.47, 0.57)*
**2022**						
1–2 people			reference	reference	reference	reference
3–4 people			1.68 (1.57, 1.80)*	1.37 (1.26, 1.49)*	0.61 (0.56, 0.66)*	0.72 (0.63 0.83)*
5 or more people			1.86 (1.68, 2.06)*	1.53 (1.34, 1.75)*	0.39 (0.34, 0.45)*	0.57 (0.46, 0.71)*

*: p < 0.05

1 OR: odds ratio; Cl = confidence interval; p = value

2 For the Tufts Equity in Health, Wealth, and Civic Engagement Study, adjusted for age, gender, race/ethnicity, educational attainment, annual household income, ZIP-code level median household income, and county- level population density. For the Household Pulse Survey, adjusted for age, gender, race/ethnicity, educational attainment, annual household income, and residence in a metropolitan statistical area. Covariates for all adjusted models determined using the evidence-based directed acyclic graph.

## Data Availability

Analytic code is available upon reasonable request from the corresponding author. Individual-level survey data for the Tufts Equity in Health, Wealth, and Civic Engagement Study may be made available upon reasonable request by email to Thomas Stopka (Thomas.Stopka@tufts.edu). All data for the Household Pulse Survey is available from https://www.census.gov/programs-surveys/household-pulse-survey/datasets.html.

## Abbreviations

aOR: Adjusted odds ratio
CI: Confidence interval
DAG: Directed acyclic graph
HEPA: High efficiency particulate air
HPS: Household Pulse Survey
NDVI: Normalized difference vegetation index
OR: Odds ratio
TES: Tufts Equity Study
